# Individual approach to long-term therapy in patients with hereditary angioedema (HAE-C1-INH): A case series

**DOI:** 10.3389/falgy.2022.949387

**Published:** 2022-08-12

**Authors:** S. Andarawewa, E. Aygören-Pürsün

**Affiliations:** Hereditary Angioedema Center, Department for Children and Adolescents, University Hospital, Goethe University, Frankfurt, Germany

**Keywords:** hereditary angioedema, long-term prophylaxis, frequency of attacks, burden of disease, angioedema control test

## Introduction

Hereditary angioedema (HAE) is caused by C1-Inhibitor (C1-INH) deficiency leading to recurrent attacks of skin swellings, abdominal pain, and laryngeal edema. HAE can cause severe disability and may be potentially life-threatening. There is considerable variation in the clinical presentation and the severity of HAE, with the frequency of attacks ranging from none to two to three times a week (1). In a recent multinational patient survey conducted before the availability of novel prophylactic regimes for HAE, the attack frequency was reported to be more than one attack per month in 51.6% of patients. Moreover, most participants (82%) showed an insufficiently controlled disease (2). Current guidelines recommend the complete control of the disease and the normalization of the patients' life as the goals of HAE treatment (3). In many cases, this implies the application of long-term prophylaxis (LTP) to prevent attacks from achieving this. The novel preventive therapies, subcutaneous and intravenous plasma-derived human C1 inhibitor concentrate (pdC1INH), lanadelumab, and berotralstat, offer safe and effective prevention of HAE attacks (3–5).

Here, we present a clinical case series of patients who were managed successfully using different types of novel long-term prophylaxis regimes.

## Methods

We assessed demographic and clinical data obtained from clinical records of three HAE-C1-INH patients with previously poorly controlled HAE. To assess disease control, we used the angioedema control test (AECT), a validated patient-reported outcome tool used in patients with recurrent angioedema. The maximum AECT score of 16 indicates complete disease control, while scores of less than 10 indicate insufficiently controlled disease (6). Type, dose, and dose intervals of the medicines for LTP as well as the frequency and characteristics of HAE-attacks prior to and break-through attacks during LTP, if any, were assessed and adverse events and tolerability were documented.

### Case 1

A 42-year-old male patient was diagnosed with hereditary angioedema with C1-INH deficiency (HAE-C1-INH) at the age of 5 years. At the age of 25 years, he presented with frequent HAE symptoms, which were predominantly abdominal pain attacks. Along with angioedema of the extremities and three episodes of laryngeal edema, the patient was treated with attenuated androgen danazol, initially in a dose of 400 mg per day for about 6 months followed by a maintenance dose of 200 mg per day for 2 years. At the time of presentation to our HAE center, the patient was experiencing three HAE attacks per month despite this therapy. These episodes usually manifested as abdominal attacks and swellings of extremities and genitals. While the patient's weight at the time of initiation of danazol LTP was 130 kg (height 183 cm, BMI 38.8), after 2 years of danazol LTP, on his first presentation at our center, his weight was 165 kg (BMI 49.3). Additionally, treatment-emergent panic attacks and anxiety were present. Danazol was discontinued, and the patient was treated with on-demand intravenous (i.v.) pdC1INH. The patient later developed psoriasis and obstructive sleep apnea, which stayed untreated due to psychological intolerability of the PAP mask and device.

When the frequency of angioedema increased to three times a week within 1 month after cessation of danazol, at the age of 33 years, LTP with pd-C1INH i.v. every 3 days, which was the available novel LTP at that time, was initiated in this patient. Nevertheless, angioedema attacks occurred once a week, requiring adaptation of the injection interval to every 2 days. However, the attack frequency remained 1–4 per month, presenting as abdominal attacks and swellings in the extremities, genitals, and buttocks.

With the approval of lanadelumab for LTP in patients with HAE, LTP with 300 mg s.c. every 14 days was initiated in this patient. Other than an abdominal attack on day 3 after starting this therapy regime, the patient remained attack-free for 7 months. The SMPC of lanadelumab allows a prolongation of the injection interval to 4 weeks (7). In this case, the interval was gradually extended up to 20 days without break-through attacks. Ten months of lanadelumab LTP were reached at this time. While receiving lanadelumab 300 mg s.c. every 22 days, three abdominal attacks occurred within 4 weeks, requiring interval readjustment to every 20 days followed by a 1-year attack-free period with this injection interval. The interval was then increased to 23 days successfully with no attacks for 4 weeks. The patient had good therapy adherence. The overall observation period under lanadelumab prophylaxis was 33 months.

The treatment was well tolerated with occasional injection site reactions.

The mean attack rate during the 3 months before initiating lanadelumab s.c. prophylaxis was 1.33 attacks per month, while during prophylaxis in the lanadelumab steady state, it was 0.09 attacks per month. This patient showed an attack reduction of 95.48% with his adaptive prophylaxis regime. Disease control was complete as assessed by AECT 16/16 at 33 months after starting prophylaxis. AECT was not available prior to prophylaxis in this patient.

### Case 2

A girl who is currently 13 years old was diagnosed with HAE-C1-INH at the age of 2 years due to her positive family history. Her first angioedema attack was a facial swelling that occurred at the age of 2.5 years. Recurrent swelling attacks further affected her face, lips, extremities, GI tract, and larynx. On-demand therapy with pdC1INH to treat attacks was administered by health care personnel. Treatment of these attacks was often delayed due to nocturnal attacks and hospitalization, which caused unnecessary diagnostic procedures despite known HAE before treatment was given. Many days of absence from school was the consequence of these unnecessary procedures and delayed therapy. After a period of reluctance to be trained on i.v. injection of C1-INH, the patient and her parents were successfully trained to self-administer subcutaneous (s.c.) icatibant to treat attacks. However, following treatment of HAE-attacks with s.c. icatibant, she regularly experienced reattacks. This patient reported 9 days of absence from school within 6 months due to HAE shortly before starting long-term prophylaxis. Angioedema attacks occurred 3–4 times a month including two laryngeal attacks. After discussing the approved long-term prophylactic regimes for this age group with the then 12-year-old patient and her parents, it was jointly resolved to start treatment with a subcutaneous C1-inhibitor. In clinical practice, doses applied may deviate from approved doses. The initial dose administered by the patient was 24 U/kg bw twice weekly. Except for an abdominal attack on day 4, the patient remained attack-free with this low-dose regime for 12 months. Dose adaptation was discussed with the patient and her caregivers after two abdominal attacks occured in the following three months, probably due to a weight gain of about 10 kg. The patient showed good compliance with the therapy.

In comparison (printing mistake) this patient experienced 2.67 attacks per month within the 3 months prior to long-term prophylaxis compared to 0.13 attacks per month during the steady state with this well-tolerated prophylactic treatment. The reduction of attack rate with the low dose was 95%***.*** The disease was poorly controlled prior to LTP with an AECT score of 4/16 and progressed to controlled disease during this prophylactic regime (AECT 14/16 at 3 months, 16/16 at 7 months, and 14/16 at 15 months of pdC1INH prophylaxis).

### Case 3

A 79-year-old female was diagnosed with HAE-C1-INH at the age of 40 years despite her first manifestation being at the age of 14 years. Initially, this patient experienced recurrent angioedema of the extremities, face, abdomen, genitals, and urinary tract, as well as signs of laryngeal edema. Following HAE diagnosis, the patient was treated with 300 mg danazol per day for prophylaxis of HAE attacks for about 17 years. Under this treatment, the patient was not free of angioedema attacks, and as a side effect, she experienced amenorrhea. After discontinuation of danazol, there was an increase in attack rate up to 2-3 times per week.

At the time of her first visit to our clinic, the patient was 66 years old, and she was treating her attacks with on-demand C1-inhibitor concentrate intravenously. With gradually decreasing vision due to macular degeneration and difficult venous access, intravenous and subcutaneous self-injection became increasingly difficult for the patient. Therefore, treatment of HAE attacks was often delayed, resulting in delayed complete remission of attacks. With HAE attacks, predominantly abdominal, occurring once a week and poor disease control and loss of vision, long-term prophylaxis with berotralstat was initiated by joint decision, as oral administration was the preferred and optimal route of administration for this patient. Berotralstat 150 mg once daily was administered and observed for a total period of 6 months. Within the observation period, there was a 2-week interruption of berotralstat intake, 4 weeks after the beginning of the prophylaxis, due to nonavailability of the drug, during which the patient experienced two HAE attacks. Following a restart with a regular intake of berotralstat 150 mg once daily, the patient was attack-free for a further observation period of 18 weeks. Good therapy adherence was apparent. Attack reduction was 100% during the regular daily intake of berotralstat 150 mg per os, and the therapy was well tolerated. Disease control moved from poorly controlled (AECT score of 8/16) before prophylaxis to fully controlled (AECT 16/16) at 24 weeks after the beginning of LTP.

## Discussion

Hereditary angioedema with C1 inhibitor deficiency (HAE-C1INH) is a rare, disabling, and potentially life-threatening disease. Symptoms range from skin swelling to mucosal swelling of the gastrointestinal system and the upper airways. Due to HAE, patients experience a complex range of physical, psychological, and social impacts (3, 8, 9). For instance, HAE patients show high levels of anxiety and depression (9). Frequent hospitalization and absence from work or school may cause an increased burden of disease not only for the patients but also for their immediate caregivers (8, 9). A substantial burden of disease remains for HAE patients despite introducing new therapies for acute attacks (10). Currently, apart from medicines to treat attacks of HAE, there are several novel, safe, and effective therapy options available for the LTP for patients with HAE to optimize HAE management (3, 4).

This report summarizes observational clinical data of three HAE-C1-INH patients in whom successful long-term prophylaxis was achieved by different therapeutic approaches (Table 1). By mutual decision between the physician and patient, individualized approaches to LTP were chosen, considering the clinical needs and preferences of the patients. LTP was further tailored individually when needed. Including the patient in the joint decision on the management of the disease may support therapy adherence, which was optimal in these three patients. The overall goal of HAE management is to achieve total disease control and normalize patients’ lives (4). In the cases presented, this goal was achieved by using LTP of different types with individual adaptations.

**Table 1:** Demographics, clinical characteristics, and treatment outcomes.

	Case 1	Case 2	Case 3
Age	42 years	13 years	79 years
Gender	Male	Female	Female
Age at the onset of HAE symptoms	3 years	2,5 years	14 years
Diagnosis of HAE	5 years	2 years	40 years
Past medical history	Hypertension, atrial fibrillation, bronchial asthma, obstructive sleep apnea, chronic gastritis, psoriasis, obesity (BMI 49,3)	Umbilical hernia operation in the first year of life	Hypertension, macular degeneration. (current vision <0,2), past history of breast cancer, past history of Hepatitis C infection
Current type and dose of LTP	Lanadelumab 300 mg s.c. every 23 days	C1-inhibitor concentrate 20U/kg bw s.c. 2x/week	Berotralstat 150 mg p.o. per day
Observation period of current LTP	33 months	15 months	6 months
Previous therapy	PdC1INH i.v. every 2 days	On-demand pdC1INH i.v.	On-demand pdC1INH i.v.
Mean attack rate per month in the 3 months before the current LTP	1.33 (under C1-INH 1,000 U i.v. every 2 days)	2.67	4.33
Mean attack rate per month during LTP during the steady state	0.09	0.13	0^a^
Percentage of attack reduction during LTP	95.48%	95.13%	100%
AECT prior to LTP	Not available	4/16	8/16
AECT with current LTP	16/16	14/16	16/16

^a^ Excluding two attacks that occurred during the 2 weeks when the patient missed her berotralstat LTP.

In case 1, frequent HAE attacks occurred previously despite i.v. C1-INH LTP administered every 2 days, which necessitated an alternative approach to LTP. As long administration intervals were of paramount interest for this patient, the decision was made to initiate LTP with lanadelumab, a monoclonal antibody against plasma kallikrein that is applied every 2 weeks. After reaching the attack-free status, doses of lanadelumab may be applied every 4 weeks (7). In the case presented, however, this interval would not have led to the prevention of attacks as this patients’ maximum interval tolerated without attacks was 23 days. With this injection interval, however attack-free status and total control of the disease could be reached.

Case 2 experienced frequent and severe HAE attacks starting early in life. Familiarity with C1-INH reassured this patient and her parents in the decision-making for LTP with SCpdC1INH. Successful LTP could be achieved with low doses of SCpdC1INH, as demonstrated by attack-free status, total control of the disease and elimination of hospitalization and absence from school due to HAE.

Severe burden of disease and patient comorbidities played a major role in the shared decision-making for LTP in case 3. The patients’ choice of mode of administration was the oral route that was also commanded by her loss of vision. Other factors that might have influenced the decision for oral prophylaxis like impaired motor skills or coordination, tremor, or needle phobia were not present in this patient. The use of an oral kalikrein inhibitor for LTP led to an immediate and enduring attack-free status and eventually to a totally controlled disease in this patient.

In all three patients, attacks were reduced by 95–100% compared to prior therapy (Table 1). Also, disease control was improved moving patients from poorly controlled to well-controlled disease with individually adapted LTP.

In summary, a customized approach for each of the patients could be found that led to optimal outcomes.

## Conclusion

This case series gives an indication of how HAE patients with a need for long-term prophylaxis may be approached to find the individually best solution. Apart from the efficacy and safety of the various types of approved LTP, the mode and frequency of administration, potential experience with the medicinal product, and pre-existing comorbidities might affect the choice of LTP. These may include impairment of sight, disturbed motor skills, or impaired coordination among others. Shared decision-making considering patients’ preference may help optimizing therapy adherence and thereby the outcome of long-term prophylaxis.

## Contribution to the field

Hereditary angioedema (HAE) due to C1 inhibitor deficiency is a rare inherited disorder that may cause recurrent swellings of the skin and gastrointestinal tract that may lead to considerable morbidity and potentially lethal upper airway edema. The frequency and severity of HAE attacks can vary from patient to patient and even within an individual over time, ranging from no attacks at all to two to three attacks per week. Therapies for patients with HAE-C1-INH to treat acute attacks have existed for many years, and effective and safe novel therapies to prevent attacks by long-term prophylaxis (LTP) have additionally become available in recent years.

Particularly, the long-term prophylactic regimes require considerable cooperation by the patient; hence, shared decision-making prepares the ground for suitable therapy adherence to ensure a good clinical outcome.

We discuss three patients with previously poorly controlled diseases, who were managed successfully using different types of novel prophylaxis regimes and therapy alterations in two cases. This case series indicates how HAE patients with a need for long-term prophylaxis may be approached to find the individually best solution. Apart from the efficacy and safety of the various types of approved LTP, the mode and frequency of adminstration, experience over medicinal products, and pre-existing comorbidities might affect the choice of LTP. These may include impairment of sight, disturbed motor skills, or impaired coordination, among others.

## Data Availability

The raw data supporting the conclusions of this article will be made available by the authors, without undue reservation.
